# Mode and Structure of the Bacterial Community on Human Scalp Hair

**DOI:** 10.1264/jsme2.ME19018

**Published:** 2019-06-20

**Authors:** Kota Watanabe, Eiji Nishi, Yukihiro Tashiro, Kenji Sakai

**Affiliations:** 1 Laboratory of Soil and Environmental Microbiology, Division of Systems Bioengineering, Department of Bioscience and Biotechnology, Faculty of Agriculture, Graduate School, Kyushu University Motooka 744, Nishi-ku, Fukuoka 819–0395 Japan; 2 Forensic Science Division, Department of Criminal Investigation, Oita Prefectural Police HQ 3–1–1 Otemachi, Oita 870–8502 Japan; 3 Laboratory of Microbial Environmental Protection, Tropical Microbiology Unit, Center for International Education and Research of Agriculture, Faculty of Agriculture, Kyushu University Motooka 744, Nishi-ku, Fukuoka 819–0395 Japan

**Keywords:** human scalp hair, bacterial community structure, scanning electron microscopy, quantitative PCR

## Abstract

Bacterial communities on various parts of the human body are distinct. We were the first to report the existence of a stable bacterial community on human scalp hair and demonstrated that an analysis of its structure by terminal restriction fragment length polymorphism (T-RFLP) is helpful for individual discrimination. However, the ecology of the bacterial community on human scalp hair has not yet been elucidated in detail. We herein investigated the mode, quantity, and phylogeny of bacterial communities on the human hair shaft and root and showed the results obtained from one representative individual. Direct SEM observations of hair, without a pretreatment, confirmed the ubiquitous presence of bacteria-like coccoids and rods on the shaft and root of hair from the human scalp, with 10^5^–10^6^ cells cm^−2^ of hair and 10^7^ cells cm^−2^ of hair, respectively. These values corresponded to the 16S rRNA gene copy numbers obtained by qPCR. These numbers were not significantly affected by detergent washing. These results represented those obtained from many individuals with different hair lengths, ages, and gender. The major OTUs on the human scalp hair shaft and root were the same and included two species of *Pseudomonas* (phylum *Proteobacteria*), *Cutibacterium* and *Lawsonella* (phylum *Actinobacteria*), and *Staphylococcus* (phylum *Firmicutes*). These results suggest that major bacteria on the human hair shaft are indigenous and derived from the hair root.

The human microbiome has been actively studied with the aims of characterizing its structure and elucidating its role in host health (28, 30). Previous studies that characterized the human microbiomes of skin (8, 13, 14), the gut (19), oral cavity (27), and vagina (24) by a 16S amplicon analysis revealed that bacterial community structures were specific for each body site and individual (11). Furthermore, relationships have been reported between the microbiomes of the gut (23) and oral cavity (36) and human disease.

Culture-dependent approaches have been widely used to isolate targeted bacteria and analyze bacterial community structures (16, 25). However, culture-dependent approaches are considered to detect less than 1% of all bacteria in the general natural environment, and, thus, may only have the ability to identify a portion of the bacterial community (1). Similarly, the number of bacterial species that may be cultured on human skin currently remains unknown, although culture-dependent approaches have been reported (12). Therefore, culture-independent approaches have become more commonly used to analyze bacterial communities. A high-throughput 16S rRNA gene analysis using next-generation sequencing (NGS), also referred to as 16S amplicon sequencing, has been recognized as a powerful and high-resolution method that shows not only the diversity of the bacterial community structure from mega-sequence data, but also phylogenetic information for major and minor bacteria (15).

There are several skin sites with human hair, including the scalp, pubis, armpits, and legs. The bacterial community structures of scalp hair have been reported in previous studies (29, 33). Tridico *et al*. analyzed bacterial community structures on pubic and scalp hair shafts using a 16S amplicon analysis and showed that two families in *Firmicutes*, *Corynebacteriaceae* and *Tissierellacea*, were predominant on scalp hair (29). However, they focused on differences in the community structures on pubic hair between sexes. In contrast, we previously reported a specific individual bacterial community structure on the roots and shafts of scalp hairs by terminal restriction fragment length polymorphism (T-RFLP); however, this method does not provide precise phylogenic information on the community structure (21). Quantitative PCR (qPCR) of bacteria on the shafts and roots of scalp hairs suggest the presence of an indigenous bacterial community.

To elucidate the number, mode, and phylogenetic bacterial community structures on human scalp hair, we directly observed bacterial cells on scalp hairs using a scanning electron microscope (SEM), quantified their numbers by qPCR, and analyzed bacterial community structures by the 16S amplicon sequencing of scalp hair samples from both the shafts and roots. We herein present the results obtained from a representative individual.

## Materials and Methods

### Sample information

Scalp hair samples were collected from six healthy Japanese and Chinese adults of both sexes (4 males and 2 females), ranging in age between 21 and 62 years old, who consented to take part in this study (Table 1). None of the volunteers were taking medication during the experimental period. Hair samples from a 28-year-old Japanese male (volunteer #001) were used as representative data. Samples of plucked or cut hairs were collected while wearing nitrile gloves. Hair shaft samples from three volunteers (#001, #004, and #006, nine hairs in total) were also tested for the effects of detergent on bacterial DNA recovery. Hair samples were immersed for 30 s without shaking in 100 mL of Triton X-100 (Sigma-Aldrich, St. Louis, MO, USA) solution (0.01, 0.1, and 0.5%, respectively). After immersion in the detergent, hairs were thoroughly rinsed with distilled water.

**Fig. 1:**
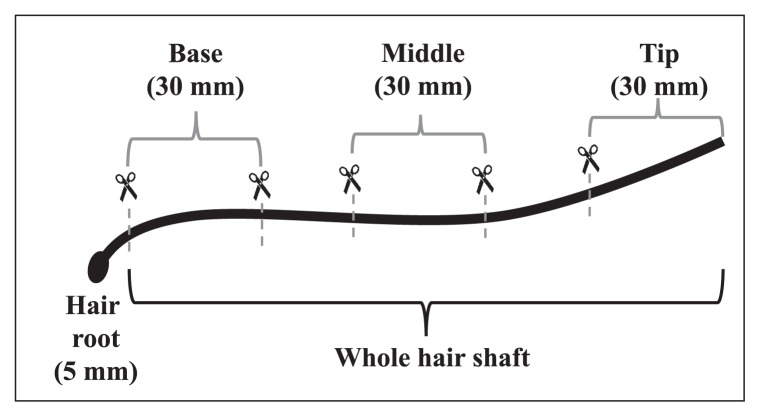
Sampling of a hair portion.

To investigate the distribution of bacteria over the length of the hair, samples were separated into four sections: the hair root and three portions (each 30 mm in length) of the hair shaft, *i.e*., the base, middle, and tip; Fig. 1). Since the hair lengths of three volunteers (#002, #003, and #005) were too short to divide into three portions, only the root and whole hair shaft were sampled. Samples from each section were obtained on at least three occasions to analyze cell numbers and bacterial community structures. This research was performed with permission from the Research Ethics Committee of the Graduate School of Bioscience and Biotechnology at Kyushu University.

### SEM observations of bacteria on scalp hair without a pretreatment under a low vacuum

We examined three scalp hairs from two volunteers (six in total). After separation into four portions, as described above, scalp hairs were attached to double-coated carbon conductive tape (Fig. 1). Scalp hairs were scanned using a SU3500 scanning electron microscope (Hitachi, Tokyo, Japan) at an acceleration voltage of 10.0–15.0 kV under a low vacuum (50 Pa) without any surface treatment, such as metal coating or dehydration. After SEM observations of scalp hair, the density of bacterial cell-like objects was assessed in three different micrographs of each portion of each sample.

### Extraction of bacterial DNA from scalp hairs

Approximately 5-mm-long pieces from the distal end were cut with sterilized scissors and used as the hair root sample. The remaining hair shafts were divided into three portions and cut into 5-mm-long pieces with sterilized scissors. Whole hair shaft samples were prepared by cutting 5-mm-long sections from whole hair shafts without separation. Bacterial DNA was extracted using the Nucleo Spin^®^ Tissue kit (MACHEREY-NAGEL, Duren, Germany) according to the manufacturer’s instructions, with a slight modification. Chopped scalp hair samples were immersed in 100 μL of lysozyme solution (20 mg mL^−1^ lysozyme derived from egg white [Wako Pure Chemical, Osaka, Japan] in 20 mM Tris-HCl and 0.2 mM EDTA, pH 8.0) at 37°C for 30 min, as previously reported (20), and the DNA extracts (100 μL) obtained were stored at −20°C until used.

### Quantification of bacterial cells on scalp hairs by qPCR

The number of bacterial cells on the hairs of six volunteers was quantified by real-time PCR (CFX Connect System, BIO-RAD, Hercules, CA, USA) using universal primers for a portion of the bacterial 16S rRNA gene. Each 10-μL reaction mixture consisted of 2 μL of KOD SYBR qPCR Mix (TOYOBO, Osaka, Japan), 0.1 μL of each primer (357F [5′-CCT ACG GGA GGC AGC AG-3′] [10] and 518R [5′-ATT ACC GCG GCT GCT GG-3′] [31]), and 2 μL of bacterial DNA. The amplification program included an initial denaturation step at 95°C for 5 min followed by 40 cycles of denaturation at 95°C for 5 s, annealing at 64°C for 20 s, and elongation at 72°C for 20 s. The DNA extract from *Escherichia coli* DH5α was used as a standard to generate a calibration curve. After amplification, the copy numbers of the 16S rRNA genes per hair sample were calculated cm^−1^ of hair and converted to cm^−2^ of hair. The following equation was used for this calculation:

$$\text{Copies }{\text{cm}}^{-2}\;\text{of hair}=\text{qPCR copies }{\text{cm}}^{-1}\;\text{of hair}\times \text{hair diameter }(\text{cm})\times \pi$$

The diameter of scalp hair was measured using a stereoscope.

### Analysis of the bacterial community structure on scalp hair by NGS

To analyze the bacterial community structure of hairs from six volunteers using the MiSeq platform (Illumina, San Diego, CA, USA), a three-step PCR method was employed using extracted DNA samples. In first-step PCR amplification, a universal primer set for the V4 region (515f, 5′-GTG CCA GCM GCC GCG GTA A-3′ and 806r, 5′-GGA CTA CHV GGG TWT CTA AT-3′) (4) was used. The 25-μL reaction mixture consisted of 12.5 μL of the Kapa HiFi HotStart Ready Mix (Kapa Biosystems, Wilmington, MA, USA), 0.5 μL of each primer (10 pM), and 11.5 μL of extracted bacterial DNA. The amplification program included an initial denaturation step at 95°C for 3 min followed by 40 cycles of denaturation at 98°C for 30 s, annealing at 56°C for 30 s, and elongation at 72°C for 30 s. After electrophoresis through a 1.5% (w/v) agarose gel, the targeted bands were excised from the gel with sterilized cutters, and DNA was extracted using the FastGene™ Gel/PCR Extraction Kit (NIPPON Genetics, Tokyo, Japan), according to the manufacturer’s instructions. DNA concentrations were measured using a NanoDrop 2000 spectrophotometer (Thermo Fisher Scientific, Waltham, MA, USA). In the preparation of 16S amplicon sequencing with MiSeq, templates are given tail, adapter, and index sequences in two-step PCR. Therefore, long-tailed primers are required for preparation, which makes amplification difficult. Direct amplification in two-step PCR was not successfully performed in the present study, and we attributed this to the amount of bacterial DNA obtained from 3-cm-long hair being very small. Therefore, we performed 1^st^ PCR using the universal primer set without artificial sequences. We successfully obtained sufficient template fragments with a minimum number of reaction cycles.

In 2^nd^ PCR, a universal primer set for the V4 region of the bacterial 16S rRNA gene and tailed sequences for MiSeq sequencing were used (1-515F, 5′-TCG TCG GCA GCG TCA GAT GTG TAT AAG AGA CAG GTG CCA GCM GCC GCG GTA A-3′ and 1-806R, 5′-GTC TCG TGG GCT CGG AGA TGT GTA TAA GAG ACA GGG ACT ACH VGG GTW TCT AAT-3′) (2). The 25-μL reaction mixture consisted of 1.0 μL of each primer (5 μM), which was heat-shocked at 95°C for 5 min, 12.5 μL of the Kapa HiFi HotStart Ready Mix, 12.5 ng of the DNA of the first-step PCR amplicon, and sterilized ultrapure water. The amplification program included an initial denaturation step at 95°C for 3 min followed by 20 cycles of denaturation at 98°C for 30 s, annealing at 55°C for 30 s, and elongation at 72°C for 30 s. PCR products were purified using the FastGene™ Gel/PCR Extraction Kit according to the manufacturer’s instructions.

In third-step PCR, a primer set with flow cell adapter sequences, index sequences, and tailed sequences was used (Forward primer, 5′-AAT GAT ACG GCG ACC ACC GAG ATC TAC AC-Index sequence-TCG TCG GCA GCG TC-3′ and Reverse primer, 5′-CAA GCA GAA GAC GGC ATA CGA GAT-Index sequence-GTC TCG TGG GCT CGG-3′). The third-step PCR mixture (25 μL) comprised 12.5 μL of the Kapa HiFi HotStart Ready Mix, 0.5 μL of each primer (10 pM), and 11.5 μL of the second-step PCR amplicon. The amplification program included an initial denaturation step at 95°C for 3 min, followed by 8 cycles of denaturation at 98°C for 30 s, annealing at 55°C for 30 s, and elongation at 72°C for 30 s. After electrophoresis on a 1.5% (w/v) agarose gel, the target bands were excised with sterilized cutters, and DNA was extracted using the FastGene™ Gel/PCR Extraction Kit as described above. The DNA concentrations of third-step PCR amplicons were quantified using the Qubit dsDNA HS Assay Kit (Thermo Fisher Scientific) according to the manufacturer’s instructions. Purified PCR products from each sample were mixed, denatured, and sequenced with an Illumina MiSeq System (Illumina) using MiSeq Reagent Kit v3 (300×2 cycles with pair-ends; Illumina), according to the manufacturer’s instructions. We obtained good coverage values (>0.95) for all hair samples using the DNA extraction kit and PCR conditions described above, indicating that the results obtained contained meaningful information (Table S1). Good coverage values were calculated using QIIME 1.9.1 (3).

### Bioinformatic analysis of MiSeq sequence data

A bioinformatic analysis of sequence data was performed using Usearch V8.1.1861 (9) and QIIME 1.9.1. The index and universal sequences of each read were checked, and reads with complete index sequences were selected as valid sequences. Usearch V8.1.1861 software was used to merge pair-end reads and remove chimeric sequences. After the chimera check, reads were grouped into operational taxonomic units (OTUs) at >97% similarity. Alpha diversity (observed OTUs and Shannon index) was evaluated at a 1% OTU distance using the QIIME software package. In the taxonomy-based analysis, representative sequences for each OTU were analyzed with EzBioCloud (6).

### Accession number

Illumina raw read sequences and the top 50 most abundant OTU sequences were deposited in the DDBJ/ENA/GenBank database under BioProject ID PRJDB8176 and accession numbers LC458617–LC458666, respectively.

## Results

### Direct observation and quantification of bacterial cells on scalp hair using SEM

**Fig. 2:**
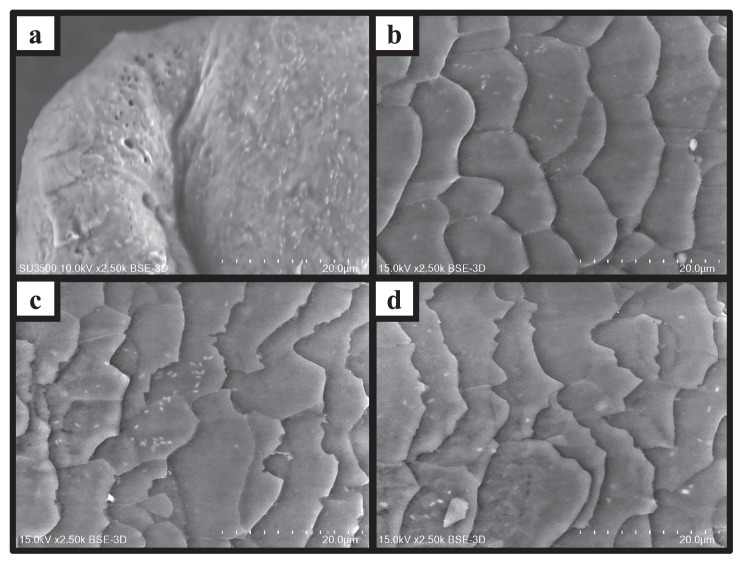
Observation of bacteria on hair by SEM. (a) The hair root, (b) base of the hair shaft, (c) middle of the hair shaft, (d) tip of the hair shaft.

Fig. 2 shows images of the surface of human scalp hair obtained by SEM without any sample pretreatment. These images showed the presence of bacteria-like rods and coccoids all over the cuticle, not only at the base, but also at the tip. The average numbers of bacteria-like cells on each site of the hair shaft were 1.0 (±0.3)×10^6^ cells cm^−2^ of hair at the base (Fig. 2b), 2.2 (±0.7)×10^6^ cells cm^−2^ of hair in the middle (Fig. 2c), and 1.6 (±0.7)×10^5^ cells cm^−2^ of hair at the tip (Fig. 2d). These results indicated that bacteria were stably attached on all sites of the scalp hair shaft. The average number of bacteria-like cells on the root was 1.3 (±0.1)×10^7^ cells cm^−2^ of hair (Fig. 2a), which was significantly higher than the number present on the shaft (ANOVA, *P*<0.05).

### Quantification of bacterial cell numbers on scalp hair by qPCR

**Fig. 3:**
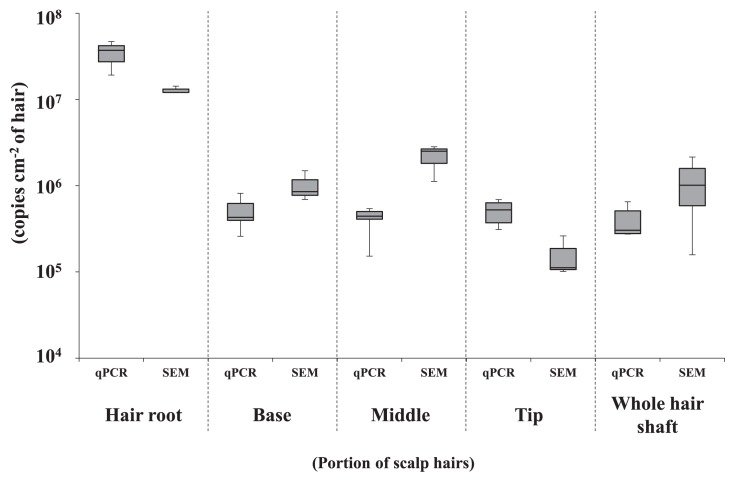
Quantification of the bacterial cell number on 5 portions of scalp hairs by qPCR of the 16S rRNA gene copy and by SEM observations.

To confirm the results of the direct SEM observations described above, quantitative PCR targeting the bacterial 16S rRNA gene was performed using DNA extracted from scalp hair samples. The values (copies cm^−2^ of hair) estimated by qPCR were compared to those (cells cm^−2^ of hair) obtained by SEM (Fig. 3). The qPCR analysis yielded similar values at each position in the three tested scalp hair samples. A strong correlation was observed between SEM and qPCR results (correlation coefficient: 0.91, *P*<0.0001). The values from the hair shaft were uniform, with an average of 5.0 (±1.9)×10^5^, 4.2 (±1.3)×10^5^, and 5.1 (±1.5)×10^5^ copies cm^−2^ of hair at the base, middle, and tip, respectively (Fig. 3). The average value at the root was 3.5 (±1.0)×10^7^ copies cm^−2^ of hair. A significantly higher number of cells was found at the hair root than on the shaft (ANOVA, *P*<0.05). The results obtained by the qPCR method corresponded well with the values obtained by the SEM method, which indicated the uniform presence of bacteria on the hair shaft.

We then measured the 16S rRNA copy number cm^−2^ of hair on scalp hairs from the five volunteers (Fig. S1). No significant differences were noted between the three portions of hair shaft from #004 and #006 (ANOVA, *P*<0.05). The values of copies cm^−2^ of hair from cells on the hair root were significantly higher than from those on the hair shaft in all volunteers (ANOVA, *P*<0.05). The copy number cm^−2^ of hair from cells on the hair root was similar among individuals, while that from cells on the hair shaft varied (10^5^–10^6^ copies cm^−2^ of hair).

### Partial 16S amplicon sequencing

In the partial 16S amplicon sequencing of 60 samples, 1,889,776 total reads were generated, yielding 1,393,788 reads after the chimera check. The average read per sample was 31,496 reads (pre-filtered) and 23,230 (post-filtered), with a range of 1,427–101,093 reads per sample (Table S1). Samples were rarefied through a core diversity analysis to 1,000 reads per sample.

### Bacterial community structures on the scalp hair shaft at the phylum level

**Fig. 4:**
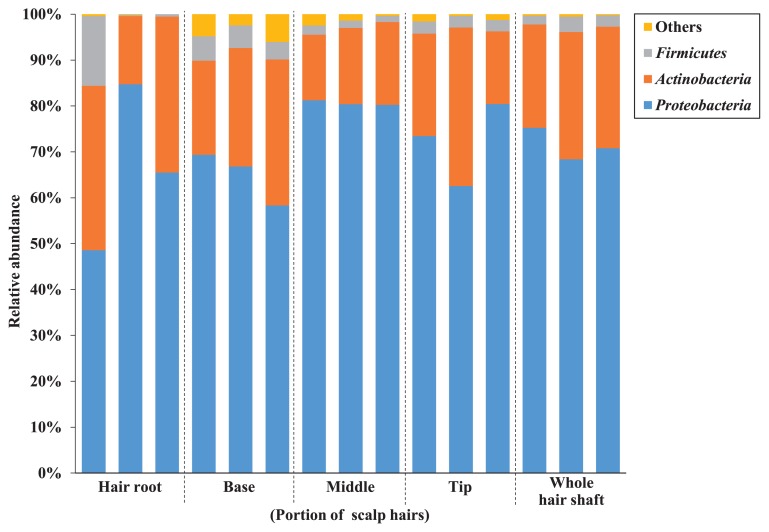
The relative abundance of major phyla in the bacterial community structure on each portion of scalp hair.

Fig. 4 shows selected representative data from a 28-year-old male, which illustrate the overall bacterial community structure in scalp hair samples from each position at the phylum level. Three major phyla on scalp hair that were common at all positions of the root and shaft were *Proteobacteria*, *Actinobacteria*, and *Firmicutes*, with average abundance of 69.7, 24.6, and 3.5%, respectively. The Kruskal-Wallis test revealed no significant differences in the abundance of these three major phyla at the different positions along the scalp hair. These results indicate that the bacterial community on scalp hair from the root to the tip had a stable structure at the phylum level in volunteer #001. Furthermore, similar structures, which included these three phyla, were observed for five other volunteers (Fig. S2).

### Alpha diversity of bacterial community structures on scalp hairs

Table 2 and S2 show the two indexes of alpha diversity, observed OTUs, and the Shannon index. The observed OTUs and Shannon index of volunteer #001 were significantly lower on the root than on the shaft. Among the three portions of the hair shaft, there was no significant difference in observed OTUs, although the base of the hair shaft showed a higher Shannon index (Table 2). A statistical analysis of the two indexes of alpha diversity in six volunteers showed that some volunteers (#001, #002, and #006) had higher observed OTUs on the hair shaft and a higher Shannon index (#001, #002, and #003) (Table 2 and S2).

### Analysis of effects of washing with detergent

The effects of washing scalp hair shafts with detergent were analyzed (Table 3 and S3), and the results obtained showed that the number of bacterial cells present before and after washing did not significantly differ. This result indicated that bacteria are tightly attached to the hair shaft. After washing, the bacterial community was still composed of the three aforementioned major phyla. The Kruskal-Wallis test revealed no significant differences in the abundance of the three major phyla among the washed hair samples. We also analyzed the observed OTUs and Shannon index of washed hair samples. Significant differences were only detected between water-rinsed and 0.5% Triton™ X-100-washed samples. These results suggested that some of the bacterial OTUs were weakly attached and susceptible to the detergent treatment.

### Bacterial community structures on scalp hairs at the species level

Based on the results obtained from one person, we assigned the top 50 most abundant OTU sequences to the most closely related bacterial species. Of these, 22 were present both at the roots and shafts, and the other 28 were found only on the shafts.

**Fig. 5:**
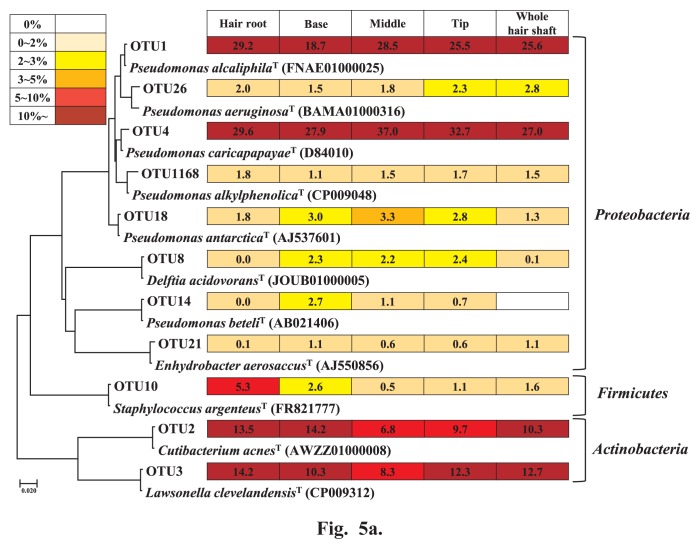

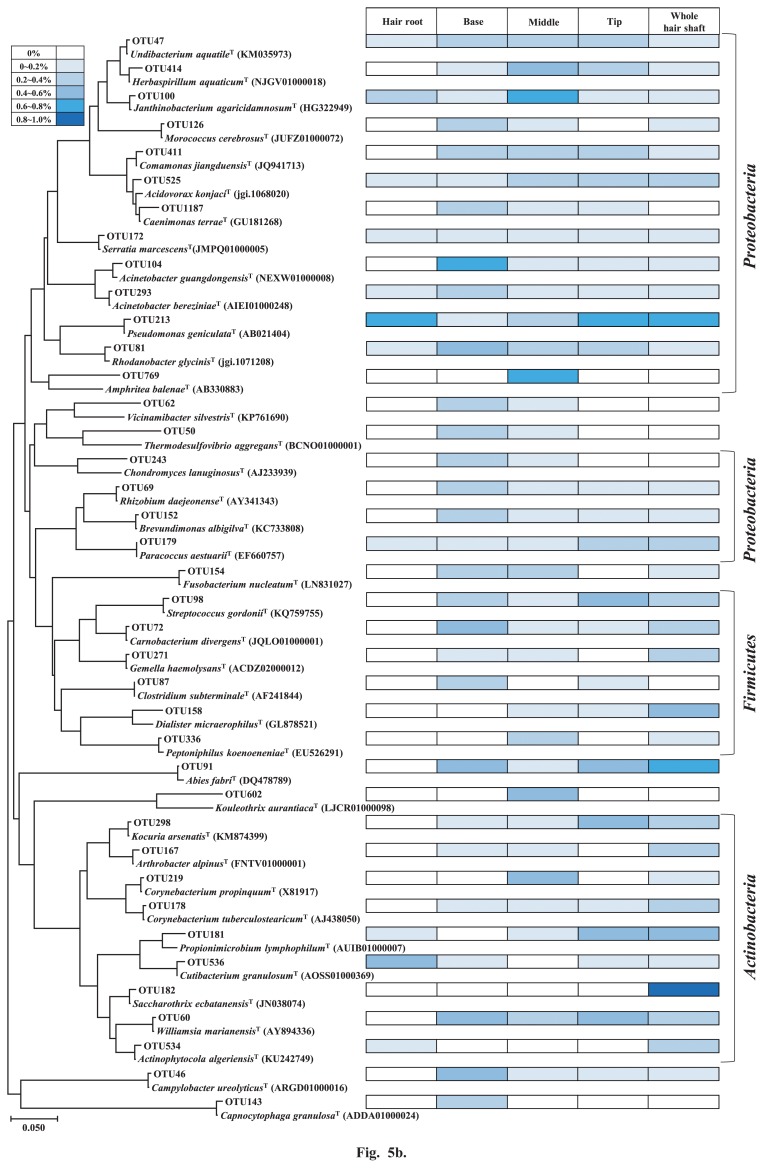
Phylogenetic tree of top 50 most abundant OTUs. The top 50 most abundant OTUs were classified into major and minor groups. Major group, OTUs show an abundance higher than 1% at any hair portion (Fig. 5a). Minor group, OTUs show an abundance less than 1% at all hair portions (Fig. 5b).

We selected 50 OTUs and classified them into two groups based on abundance: major, ≥1% relative abundance at any position (11 OTUs, Fig. 5a); and minor, ≤1% relative abundance at any position (39 OTUs, Fig. 5b). Eleven OTUs in the major group were dominant, with >90% relative abundance at each position of scalp hair. Of these, four OTUs (Phylum, relative abundance, pairwise similarity) were predominant species, with >7% relative abundance at all positions: OTU1, related to *Pseudomonas alcalophila* ( *Proteobacteria*, 18.7–28.5%, 99.7%); OTU4, related to *P. caricapapayae* (*Proteobacteria*, 27.0–37.0%, 99.0%); OTU2, related to *Cutibacterium acnes* (*Actinobacteria*, 6.8–14.2%, 99.7%); and OTU3, related to *Lawsonella clevelandensis* (*Actinobacteria*, 8.3–14.2%, 99.7%). In contrast, OTU10, related to *Staphylococcus argenteus* ( *Firmicutes*, 0.5–5.3%, 99.7%), showed higher abundance at the roots. Another six OTUs, which were related to the genera *Pseudomonas*, *Enhydrobacter*, and *Delftia* (*Proteobacteria*, 0–3.3%, 98.3–99.7%) were rare at the root; OTU8, related to *Delftia acidovorans* (*Proteobacteria*, 0.03–2.4%, 99.7%); OTU14, related to *P. beteli* (*Proteobacteria*, 0–2.7%, 99.7%); and OTU21, related to *Enhydrobacter aerosaccus* (*Proteobacteria*, 0.1–1.1%, 99.3%).

Of the 39 OTUs in the minor group, only 11 (OTU 47, 81, 100, 143, 172, 179, 181, 213, 293, 525, and 536) were present at both the roots and shafts, whereas the other 28 OTUs were not found at the hair root. This result suggested that more than half of the minor OTUs originated from outside the scalp hair, whereas the major bacteria on the hair shaft were indigenous and derived from the hair root.

## Discussion

Investigations on the human microbiome on skin showed diversity in bacterial community structures, not only between individuals, but also among body sites (8, 13, 14). Relationships have also been reported between bacterial community structures and human health (26). In contrast, limited information is currently available on the bacterial community structure on human scalp hair. In the present study, we aimed to elucidate the structure of the bacterial community on human scalp hair, both on the shaft and at the root, using SEM, qPCR, and NGS.

Direct observations by SEM revealed the ubiquitous presence of bacteria-like rods and coccoids on human scalp hairs not only at the roots, but also on the shafts (Fig. 2). To the best of our knowledge, this is the first study of bacteria-like cells on the shafts and roots of scalp hair, with an unexpectedly high density. Mase *et al*. observed scalp hair shafts by SEM after immersion in a bacterial suspension (17). Yun *et al*. reported bacterial contamination on sampled hair shafts maintained under a room air atmosphere for 1 week (35). Matard *et al*. found bacterial biofilms on natural scalp hair follicles in both patient and control groups (18).

The densities of the bacteria present on several sites of the human body have been reported, including 10^2^ cells cm^−2^ on dried skin and 10^7^ cells cm^−2^ on wet skin (12), 10^6^ cells mL^−1^ in saliva (27), and 10^10^ cells g^−1^ of the intestinal content (19). We previously showed the amount of bacterial DNA obtained from scalp hairs (both roots and shafts) (21), but not cell densities. In the present study, we quantified bacterial cell densities on scalp hairs both by counting on SEM and qPCR, revealing high densities of *ca*. 10^7^ cells cm^−2^ at the roots and 10^6^ cells cm^−2^ on the shafts (Fig. 3). It is important to note that bacterial cell density on the scalp hair shaft is of the same order as those on wet skin and in saliva (11, 27). Another important point is that bacterial densities on the shaft of scalp hair were similar from the bottom to the top in the three volunteers tested. Furthermore, the number of bacteria on the whole hair shaft from three volunteers tested did not significantly change even after washing with detergent (Fig. S1 and Table S3). The electrostatic attraction and hydrophobic properties of hair appear to contribute to robust bacterial adhesion (17, 35). Scalp hair cuticles are suspected to be favorable sites for the adhesion of bacteria. Based on their ubiquitous distribution and high density on the hair shaft, bacteria may adhere via unknown biological interactions with the scalp hair shaft.

We identified three predominant phyla on scalp hair shafts: *Proteobacteria* (avg. 53.0%), *Actinobacteria* (avg. 34.6%), and *Firmicutes* (avg. 9.6%) (Fig. 4). Similar phylum structures and abundance, which were consistent with the present results, were previously reported on hair roots (8, 29). *Actinobacteria* (71–78%) and *Firmicutes* (22–26%) are two major phyla on scalp skin (7, 32, 34). On the other hand, Perez *et al.* (22) showed that *Actinobacteria* (avg. 27%), *Firmicutes* (avg. 62%), and *Proteobacteria* (avg. 5%) are three major phyla found on scalp skin. The differences observed in dominance at the phylum level in these studies may have been due to differences in the DNA extraction methods used, such as enzyme treatments. Although a few studies have already reported structures at the family level (8, 29), we initially clarified structures at the genus and species levels. Four major OTUs commonly found on both the shafts and roots belonged to the genera *Pseudomonas*, *Cutibacterium*, *Lawsonella*, and *Staphylococcus* (Fig. 5a). This result supports our previous findings showing that major OTUs on the scalp hair shaft were indigenous and originated from the hair root. Several bacteria on skin belonging to these genera are considered to proliferate using sebum and sweat as nutrients, and their role in the degradation of lipids in human skin has been suggested (5). These major bacteria may interact with scalp hair. In contrast, minor bacterial OTUs were more varied, with more than two-thirds being found on the hair shaft only in volunteer #001.

In conclusion, we showed the ubiquitous distribution of bacterial cells on the shaft and root of human scalp hair, both by low pressure SEM and qPCR. The major bacteria detected on hair samples collected from several volunteers were common not only at the hair root, but also on the shaft. They were classified into two species of the genus *Pseudomonas* (phylum *Proteobacteria*), *Cutibacterium* and *Lawsonella* (phylum *Actinobacteria*), and *Staphylococcus* (phylum *Firmicutes*). As described above, we hypothesized that bacterial community structures on the scalp hair shaft originate not only from the surrounding environment, but also from the human scalp hair root. Further studies to investigate general and specific bacterial community structures on scalp hairs from more individuals are currently ongoing.

## SUPPLEMENTARY MATERIAL



## Figures and Tables

**Table 1:** Details of human scalp hair samples in six volunteers.

Volunteer	Gender	Age	Hair length (mm)	Hair diameter (μm)	Hair dye*	Hair wax
#001	Male	28	92 (±16)	84	−	−
#002	Male	39	37 (±6)	111.4	+	−
#003	Male	62	42 (±4)	61.8	−	−
#004	Female	35	370 (±82)	70.8	−	−
#005	Male	25	66 (±2)	87.1	−	−
#006	Female	21	348 (±98)	68.4	+	−

* Volunteers #002 and #006 had dyed their hair more than 2 weeks before sampling.

**Table 2:** Alpha diversity of hair samples based on observed OTUs and the Shannon index. These values were obtained from the clustering of 1,000 reads per sample.

Hair portion	Observed OTUs	Shannon
Base	49.8 (±11.7)^b^	3.5 (±0.3)^d^
Middle	47.2 (±4.7)^b^	2.9 (±0.04)^ab^
Tip	45.9 (±6.7)^b^	3.0 (±0.3)^b^
Whole hair shaft	52.8 (±6.2)^c^	3.2 (±0.2)^c^
Hair root	30.8 (±3.5)^a^	2.7 (±0.2)^a^

*P*<0.05

**Table 3:** Bacterial copy number, phylum level abundance, and alpha diversity after the treatment of human scalp hair.

Treatment	Copy number cm^−2^ of hair	Phylum level abundance (%)				Alpha diversity	
	
		*Proteobacteria*	*Actinobacteria*	*Firmicutes*	Others	Observed OTUs	Shannon
No treatment	7.2 (±1.6)×10^5^	70.3 (±3.7)	26.6 (±2.8)	2.2 (±0.6)	0.9 (±0.5)	57.7 (±8.3)^ab^	3.1 (±0.3)^b^
Rinse with water	7.1 (±0.6)×10^5^	64.7 (±3.4)	31.3 (±3.0)	3.0 (±1.4)	1.0 (±0.2)	57.9 (±5.7)^b^	3.1 (±0.1)^b^
Rinse with Triton X-100 (0.01%)	7.6 (±1.2)×10^5^	67.2 (±8.7)	29.3 (±7.0)	2.5 (±1.5)	0.9 (±0.4)	56.8 (±11.2)^ab^	3.1 (±0.5)^ab^
Rinse with Triton X-100 (0.1%)	8.3 (±1.4)×10^5^	76.3 (±3.8)	21.8 (±3.5)	1.5 (±0.2)	0.4 (±0.2)	53.7 (±5.8)^ab^	2.9 (±0.2)^a^
Rinse with Triton X-100 (0.5%)	9.4 (±1.4)×10^5^	78.5 (±3.8)	19.7 (±3.5)	1.5 (±0.4)	0.3 (±0.1)	51.9 (±4.7)^a^	2.8 (±0.2)^a^

*P*<0.05

## References

[1] Amann R.I., Ludwig W., Schleifer K.H. (1995). Phylogenetic identification and in situ detection of individual microbial cells without cultivation. Microbiol Rev.

[2] Brown C.T., Hug L.A., Thomas B.C. (2015). Unusual biology across a group comprising more than 15% of domain bacteria. Nature.

[3] Caporaso J.G., Kuczynski J., Stombaugh J. (2010). QIIME allows analysis of high-throughput community sequencing data. Nat Methods.

[4] Caporaso J.G., Lauber C.L., Walters W.A., Berg-Lyons D., Lozupone C.A., Turnbaugh P.J., Fierer N., Knight R. (2011). Global patterns of 16S rRNA diversity at a depth of millions of sequences per sample. Proc Natl Acad Sci USA.

[5] Chiller K., Selkin B.A., Murakawa G.J. (2001). Skin microflora and bacterial infections of the skin. J Invest Dermatol Symp Proc Proc.

[6] Chun J., Lee J.H., Jung Y., Kim M., Kim S., Kim B.K., Lim Y.-W. (2007). EzTaxon: a web-based tool for the identification of prokaryotes based on 16S ribosomal RNA gene sequences. Int J Syst Evol Microbiol.

[7] Clavaud C., Jourdain R., Bar-Hen A. (2013). Dandruff is associated with disequilibrium in the proportion of the major bacterial and fungal populations colonizing the scalp. PLoS One.

[8] Costello E.K., Lauber C.L., Hamady M., Fierer N., Gordon J.I., Knight R. (2009). Bacterial community variation in human body habitats across space and time. Science.

[9] Edgar R.C. (2010). Search and clustering orders of magnitude faster than BLAST. Bioinformatics.

[10] Fierer N., Jackson J. (2005). Assessment of soil microbial community structure by use of taxon-specific quantitative PCR assays. Appl Environ Microbiol.

[11] Fierer N., Lauber C.L., Zhou N., McDonald D., Costello E.K., Knight R. (2010). Forensic identification using skin bacterial communities. Proc Natl Acad Sci USA.

[12] Fredricks D.N. (2001). Microbial ecology of human skin in health and disease. J Invest Dermatol Symp Proc Proc.

[13] Grice E.A., Kong H.H., Conlan S. (2009). Topographical and temporal diversity of the human skin microbiome. Science.

[14] Grice E.A., Segre J.A. (2011). The skin microbiome. Nat Rev Microbiol.

[15] Levy S.E., Myers R.M. (2016). Advancements in next-generation sequencing. Annu Rev Genomics Hum Genet.

[16] Leyden J.J., McGinley K.J., Nordstrom K.M., Webster G.F. (1987). Skin microflora. J Invest Dermatol.

[17] Mase K., Hasegawa T., Horii T., Hatakeyama K., Kawano Y., Yamashino T., Ohta M. (2000). Firm adherence of *Staphylococcus aureus* and *Staphylococcus epidermidis* to human hair and effect of detergent treatment. Microbiol Immunol.

[18] Matard B., Meylheuc T., Briandet R., Casin I., Assouly P., Cavelier-Balloy B., Reygagne P. (2013). First evidence of bacterial biofilms in the anaerobe part of scalp hair follicles: a pilot comparative study in folliculitis decalvans. J Eur Acad Dermatol Venereol.

[19] Nakayama J., Watanabe K., Jiang J. (2015). Diversity in gut bacterial community of school-age children in Asia. Sci Rep.

[20] Nishi E., Tashiro Y., Sakai K. (2015). Discrimination among individuals using terminal restriction fragment length polymorphism profiling of bacteria derived from forensic evidence. Int J Legal Med.

[21] Nishi E., Watanabe K., Tashiro Y., Sakai K. (2017). Terminal restriction fragment length polymorphism profiling of bacterial flora derived from single human hair shafts can discriminate individuals. Leg Med.

[22] Perez G.I., Gao Z., Jourdaiun R., Ramirez J., Gany F., Clavaud C., Demaude J., Breton L., Blaser M.J. (2016). Body site is a more determinant factor than human population diversity in the healthy skin microbiome. PLoS One.

[23] Qin J., Li Y., Cai Z. (2012). A metagenome-wide association study of gut microbiota in type 2 diabetes. Nature.

[24] Ravel J., Gajer P., Abdo Z. (2011). Vaginal microbiome of reproductive-age women. Proc Natl Acad Sci USA.

[25] Roth R.R., James W.D. (1988). Microbial ecology of the skin. Annu Rev Microbiol.

[26] Sanford J.A., Gallo R.L. (2013). Functions of the skin microbiota in health and disease. Semin Immunol.

[27] Sedgley C.M., Nagel A.C., Shelburne C.E., Clewell D.B., Appelbe O., Molander A. (2005). Quantitative real-time PCR detection of oral *Enterococcus faecalis* in humans. Arch Oral Biol.

[28] The NIH HMP Working Group (2009). The NIH human microbiome project. Genome Res.

[29] Tridico S.R., Murray D.C., Addison J., Kirkbride K.P., Bunce M. (2014). Metagenomic analyses of bacteria on human hairs: a qualitative assessment for applications in forensic science. Invest Genet.

[30] Turnbaugh P.J., Ley R.E., Hamady M., Fraser-Liggett C.M., Knight R., Gordon J.I. (2007). The human microbiome project. Nature.

[31] Turner S., Pryer K.M., Miao V.P.W., Palmer J.D. (1999). Investigating deep phylogenetic relationships among cyanobacteria and plastids by small subunit rRNA sequence analysis. J Eukaryot Microbiol.

[32] Wang L., Clavaud C., Bar-Hen A. (2015). Characterization of the major bacterial-fungal populations colonizing dandruff scalps in Shanghai, China, shows microbial disequilibrium. Exp Dermatol.

[33] Williams D.W., Gibson G. (2017). Individualization of pubic hair bacterial communities and the effects of storage time and temperature. Forensic Sci Int: Genet.

[34] Xu Z., Wang Z., Yuan C. (2016). Dandruff is associated with the conjoined interactions between host and microorganisms. Sci Rep.

[35] Yun A., Yang E.J., Lee Y.M., Chae S.S., Seo H.N., Park D.H. (2010). Quantitative and qualitative estimation of bacteria contaminating human hairs. J Bacteriol Virol.

[36] Zheng H., Xu L., Wang Z., Li L., Zhang J., Zhang Q., Chen T., Lin J., Chen F. (2015). Subgingival microbiome in patients with healthy and ailing dental implants. Sci Rep.

