# Supplementary material for: Mode and Structure of the Bacterial Community on Human Scalp Hair
Source: Microbes Environ. 2019 Jun 20;34(3):252–9. doi: 10.1264/jsme2.ME19018 (PMC6759350; doi:10.1264/jsme2.ME19018)
Supplement: Supplementary file 1 [file 34_252_s1.pdf]

Fig. S1 Watanabe et al.

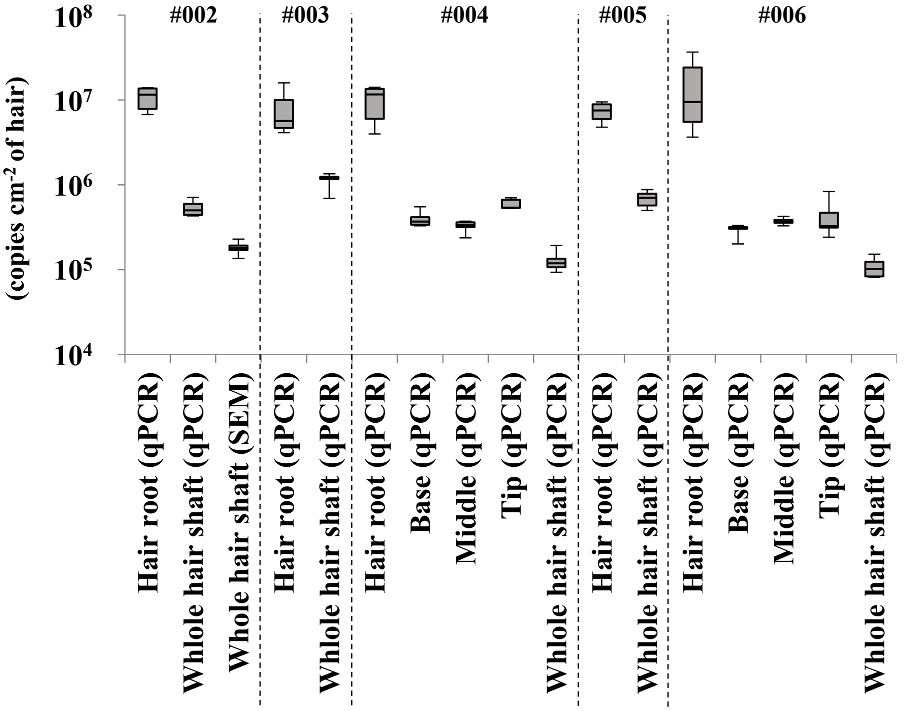

Fig. S2 Watanabe et al.

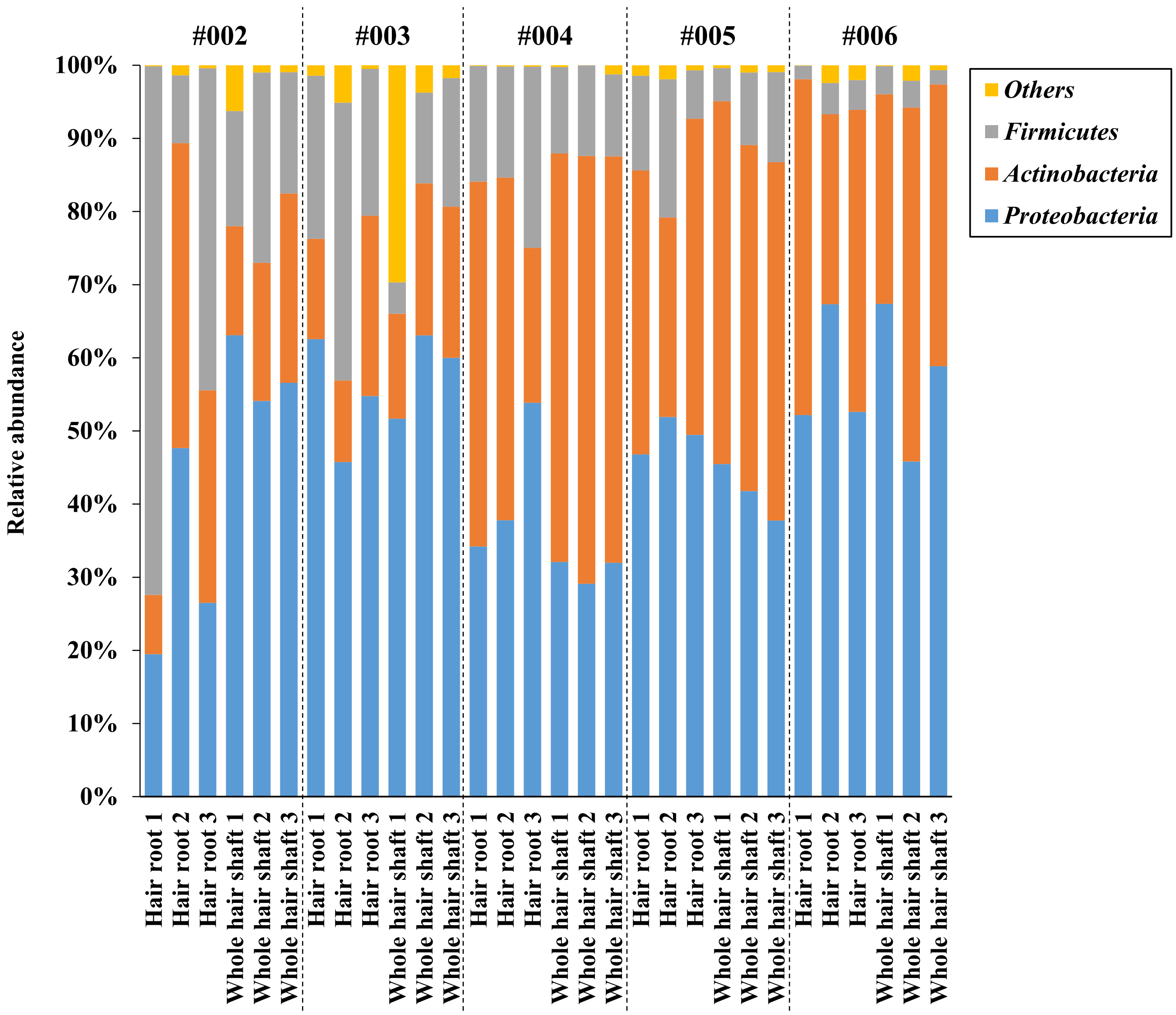

Table S1 Watanabe et al.

Sample number	Volunteer ID	Gender	Age	Portion	Treatment	Hair length (mm)	Read number (pre-filtered)	Read number (post-filtered)	Good coverage value (%)
1	#001	Male	28	Hair root	Non treatment	-	17,422	4,762	98.5
2	#001	Male	28	Hair root	Non treatment	-	10,647	3,389	99.0
3	#001	Male	28	Hair root	Non treatment	-	30,848	11,216	99.0
4	#001	Male	28	Base	Non treatment	30	20,941	17,733	98.2
5	#001	Male	28	Base	Non treatment	30	7,205	5,951	98.8
6	#001	Male	28	Base	Non treatment	30	50,114	42,690	98.5
7	#001	Male	28	Middle	Non treatment	30	1,999	1,666	97.1
8	#001	Male	28	Middle	Non treatment	30	5,416	4,503	98.4
9	#001	Male	28	Middle	Non treatment	30	9,910	8,464	98.5
10	#001	Male	28	Tip	Non treatment	30	49,690	38,437	98.6
11	#001	Male	28	Tip	Non treatment	30	1,702	1,427	97.7
12	#001	Male	28	Tip	Non treatment	30	2,781	2,469	98.6
13	#001	Male	28	Whole hair shaft	Non treatment	111	26,140	6,785	98.0
14	#001	Male	28	Whole hair shaft	Non treatment	117	24,264	5,578	97.4
15	#001	Male	28	Whole hair shaft	Non treatment	131	21,671	4,825	97.8
16	#001	Male	28	Whole hair shaft	Non treatment	79	167,285	101,093	97.9
17	#001	Male	28	Whole hair shaft	Non treatment	81	41,786	23,778	97.9
18	#001	Male	28	Whole hair shaft	Non treatment	83	36,876	21,827	97.4
19	#001	Male	28	Whole hair shaft	Water	70	6,449	3,812	97.7
20	#001	Male	28	Whole hair shaft	Water	81	79,113	45,418	97.6
21	#001	Male	28	Whole hair shaft	Water	82	96,465	58,300	98.0
22	#001	Male	28	Whole hair shaft	0.01% Triton	78	17,047	9,817	98.1
23	#001	Male	28	Whole hair shaft	0.01% Triton	100	95,930	56,653	97.3
24	#001	Male	28	Whole hair shaft	0.01% Triton	105	29,534	17,883	97.8
25	#001	Male	28	Whole hair shaft	0.1% Triton	77	11,360	6,166	97.8
26	#001	Male	28	Whole hair shaft	0.1% Triton	98	21,664	11,855	97.7
27	#001	Male	28	Whole hair shaft	0.1% Triton	100	23,927	12,638	97.6
28	#001	Male	28	Whole hair shaft	0.5% Triton	85	34,496	19,175	98.0
29	#001	Male	28	Whole hair shaft	0.5% Triton	88	18,356	9,366	97.6
30	#001	Male	28	Whole hair shaft	0.5% Triton	95	10,413	6,234	98.1
31	#002	Male	39	Hair root	Non treatment	-	32,642	29,103	98.7
32	#002	Male	39	Hair root	Non treatment	-	7,385	6,796	98.9
33	#002	Male	39	Hair root	Non treatment	-	30,100	27,041	98.6
34	#002	Male	39	Whole hair shaft	Non treatment	30	27,831	25,742	98.9
35	#002	Male	39	Whole hair shaft	Non treatment	35	40,109	36,982	99.2
36	#002	Male	39	Whole hair shaft	Non treatment	45	29,741	24,315	99.2
37	#003	Male	62	Hair root	Non treatment	-	36,207	33,654	99.2
38	#003	Male	62	Hair root	Non treatment	-	37,474	35,030	99.2
39	#003	Male	62	Hair root	Non treatment	-	26,787	24,716	98.8
40	#003	Male	62	Whole hair shaft	Non treatment	37	48,909	44,908	97.9
41	#003	Male	62	Whole hair shaft	Non treatment	42	49,243	46,048	98.8
42	#003	Male	62	Whole hair shaft	Non treatment	47	44,974	42,083	98.8
43	#004	Female	35	Hair root	Non treatment	-	23,163	20,893	99.0
44	#004	Female	35	Hair root	Non treatment	-	30,317	27,270	98.5
45	#004	Female	35	Hair root	Non treatment	-	14,026	6,050	99.1
46	#004	Female	35	Whole hair shaft	Non treatment	270	42,845	39,947	99.0
47	#004	Female	35	Whole hair shaft	Non treatment	370	35,877	33,712	99.1
48	#004	Female	35	Whole hair shaft	Non treatment	470	29,472	27,303	98.6
49	#005	Male	25	Hair root	Non treatment	-	34,816	29,911	98.5
50	#005	Male	25	Hair root	Non treatment	-	25,205	22,933	99.2
51	#005	Male	25	Hair root	Non treatment	-	19,410	17,221	98.6
52	#005	Male	25	Whole hair shaft	Non treatment	63	27,171	25,136	99.0
53	#005	Male	25	Whole hair shaft	Non treatment	65	49,064	45,032	98.9
54	#005	Male	25	Whole hair shaft	Non treatment	69	37,443	34,675	98.5
55	#006	Female	21	Hair root	Non treatment	-	25,322	21,514	98.6
56	#006	Female	21	Hair root	Non treatment	-	42,275	37,048	98.7
57	#006	Female	21	Hair root	Non treatment	-	14,984	13,071	99.0
58	#006	Female	21	Whole hair shaft	Non treatment	212	23,265	21,592	98.4
59	#006	Female	21	Whole hair shaft	Non treatment	395	9,732	9,234	98.1
60	#006	Female	21	Whole hair shaft	Non treatment	437	22,536	20,918	98.2

Table S2 Watanabe et al.

Volunteer	Portion of hair	Observed OTUs	Shannon
#002	Whole hair shaft	42.6(±2.5)[b]	3.8(±0.2)[b]
#002	Hair root	38.0(±5.3)[a]	3.2(±0.2)[a]
#003	Whole hair shaft	30.3(±11.5)[b]	4.3(±0.3)[b]
#003	Hair root	42.6(±3.8)[a]	3.9(±0.1)[a]
#004	Whole hair shaft	40.0(±7.0)[a]	3.2(±0.2)[a]
#004	Hair root	41.8(±5.2)[a]	3.6(±0.3)[b]
#005	Whole hair shaft	46.8(±7.8)[a]	3.5(±0.3)[a]
#005	Hair root	47.5(±6.8)[a]	3.7(±0.2)[b]
#006	Whole hair shaft	51.4(±4.0)[b]	3.3(±0.3)[a]
#006	Hair root	39.8(±7.5)[a]	3.2(±0.4)[a]

Table S3 Watanabe et al.

Volunteer	Treatment	Copy number/ cm^2-hair
#004	Non-treatment	$1.3 (\pm 0.3) \times 10^5$
	Rinse with water	$7.7 (\pm 0.9) \times 10^4$
	Rinse with Triton X-100 (0.01%)	$7.9 (\pm 2.3) \times 10^4$
	Rinse with Triton X-100 (0.1%)	$1.3 (\pm 0.5) \times 10^5$
	Rinse with Triton X-100 (0.5%)	$1.0 (\pm 0.2) \times 10^5$
#006	Non-treatment	$1.1 (\pm 0.3) \times 10^5$
	Rinse with water	$9.8 (\pm 0.6) \times 10^4$
	Rinse with Triton X-100 (0.01%)	$7.3 (\pm 2.0) \times 10^4$
	Rinse with Triton X-100 (0.1%)	$9.7 (\pm 1.0) \times 10^5$
	Rinse with Triton X-100 (0.5%)	$8.8 (\pm 1.4) \times 10^4$

Tab. S1 Details of hair samples for MiSeq analysis.

Tab. S2 Alpha diversity (observed OTU and the Shannon index) of the hair samples in five volunteers. The values are obtained from clustering of 1,000 reads per sample.

Tab. S3 Bacterial copy number after various treatments of human scalp hair in two volunteers.

Fig. S1 Quantification of the bacterial cell number on scalp hair by qPCR of 16S rRNA gene copy and by SEM observation in five volunteers.

Fig. S2 The relative abundance of the major phyla of bacterial community structure on scalp hair root and shaft in five volunteers.
